# Olfactory receptors are expressed in pancreatic β-cells and promote glucose-stimulated insulin secretion

**DOI:** 10.1038/s41598-018-19765-5

**Published:** 2018-01-24

**Authors:** Yuichiro Munakata, Tetsuya Yamada, Junta Imai, Kei Takahashi, Sohei Tsukita, Yuta Shirai, Shinjiro Kodama, Yoichiro Asai, Takashi Sugisawa, Yumiko Chiba, Keizo Kaneko, Kenji Uno, Shojiro Sawada, Hiroyasu Hatakeyama, Makoto Kanzaki, Jun-ichi Miyazaki, Yoshitomo Oka, Hideki Katagiri

**Affiliations:** 1 Department of Metabolism and Diabetes, Tohoku University Graduate School of Medicine, 2-1 Seiryo-machi, Aoba-ku Sendai, 980-8575 Japan; 2 Center for Metabolic Diseases, Tohoku University Graduate School of Medicine, Sendai, 980-8575 Japan; 3 Graduate School of Biomedical Engineering, Tohoku University, Sendai, 980-8579 Japan; 4 Division of Stem Cell Regulation Research, Osaka University Graduate School of Medicine, Suita, 565-0871 Japan; 5 Japan Agency for Medical Research and Development (AMED), CREST, Chiyoda-ku Tokyo, 100-0004 Japan

## Abstract

Olfactory receptors (ORs) mediate olfactory chemo-sensation in OR neurons. Herein, we have demonstrated that the OR chemo-sensing machinery functions in pancreatic β-cells and modulates insulin secretion. First, we found several OR isoforms, including OLFR15 and OLFR821, to be expressed in pancreatic islets and a β-cell line, MIN6. Immunostaining revealed OLFR15 and OLFR821 to be uniformly expressed in pancreatic β-cells. In addition, mRNAs of *Olfr15* and *Olfr821* were detected in single MIN6 cells. These results indicate that multiple ORs are simultaneously expressed in individual β-cells. Octanoic acid, which is a medium-chain fatty acid contained in food and reportedly interacts with OLFR15, potentiated glucose-stimulated insulin secretion (GSIS), thereby improving glucose tolerance *in vivo*. GSIS potentiation by octanoic acid was confirmed in isolated pancreatic islets and MIN6 cells and was blocked by OLFR15 knockdown. While *Gα*_*olf*_ expression was not detectable in β-cells, experiments using inhibitors and siRNA revealed that the pathway dependent on phospholipase C-inositol triphosphate, rather than cAMP-protein kinase A, mediates GSIS potentiation via OLFR15. These findings suggest that the OR system in pancreatic β-cells has a chemo-sensor function allowing recognition of environmental substances obtained from food, and potentiates insulin secretion in a cell-autonomous manner, thereby modulating systemic glucose metabolism.

## Introduction

The olfactory system has an important function as an environmental sensor. This system is used to find food, detect predators and mark territory^1^. Olfactory receptors (ORs) are expressed in OR neurons (ORNs), where they detect exogenous chemical ligands, referred to as odorants^2^. ORs form the largest receptor family in mammals, consisting of 1037 and 388 putatively functional ORs in mice and humans, respectively^3,4^. It is well known that, in mammalian ORNs, only one OR isoform is expressed in one neuron and projections from ORNs expressing the same ORs converge exclusively on one or two glomeruli in the olfactory bulb^5,6^. This multilayer mechanism leads to recognition of divergent and overlapping odor information.

Microarray and deep sequencing analyses have recently demonstrated that ORs are expressed in many tissues other than ORNs^7,8^, but little is known about ectopic OR functions. Human olfactory receptor 17-4 (hOR17-4) and mouse olfactory receptor 23 (MOR23) expressed in human and murine spermatozoa, respectively, reportedly function in sperm chemotaxis^9,10^, while MOR23 is also present in skeletal muscle and has a role in skeletal muscle regeneration^11^. In addition, OLFR78 expressed in the carotid body and the kidney reportedly plays a role in the regulation of breathing^12^ and blood pressure^13^, respectively. Pancreatic β-cells, which secrete insulin to regulate systemic glucose metabolism, receive nutritional information by sensing changes in circulating substances which reflect digested food. We speculated that this might be conceptually analogous to olfactory perception, although, to our knowledge, there have been no studies examining the OR system in pancreatic β-cells. Therefore, we focused on the involvement of the OR system in the secretion of insulin from pancreatic β-cells.

Insulin secretion is well known to be markedly enhanced by glucose, a phenomenon referred to as glucose-stimulated insulin secretion (GSIS). GSIS plays a major role in lowering postprandial glucose levels and thereby maintaining glucose homeostasis. In addition to glucose, other nutrients, such as free fatty acids (FFAs), amino acids and fructose also modulate insulin secretion^14–17^. Therefore, we hypothesized that an ectopic OR system in pancreatic β-cells, if present, would have a chemo-sensor function allowing sensing of circulating substances which reflect digested food and would be involved in regulating insulin secretion.

First, we performed a microarray analysis to comprehensively examine mRNA expressions in pancreatic islets and a pancreatic β-cell line, MIN6, and obtained data suggesting several ORs, including *Olfr15*, to be ectopically expressed in pancreatic β-cells. In a previous study designed to identify ligands for a large number of mammalian ORs, many odorant-receptor interactions were examined by high-throughput screening using the luciferase assay system in 293 T cells overexpressing the olfaction-specific G protein (Gα_olf_), multiple chaperones and OR together^18^. OLFR15, a mouse olfactory receptor, was shown to interact with several substances. Among them, octanoic acid (OA) is a medium-chain fatty acid (MCFA) contained in natural food products, and reportedly enhances GSIS from the perfused rat pancreas^19^, although it remains unclear whether OA is a functional ligand for OLFR15. Based on these findings, we focused on OLFR15 in the present study and clearly showed OLFR15 to be functionally expressed in pancreatic β-cells. Interestingly, our results suggest that OLFR15 plays an important role in modulating insulin secretion via the phospholipase C (PLC)-inositol triphosphate (IP_3_)-dependent pathway. Thus, the OR system exists in pancreatic β-cells and functions to directly regulate insulin secretion in a cell-autonomous manner.

## Results

### OLFR15 and OLFR821 are simultaneously expressed in almost every pancreatic β-cell, and OLFR15 is expressed on the plasma membranes of pancreatic β-cells

**Figure 1:**
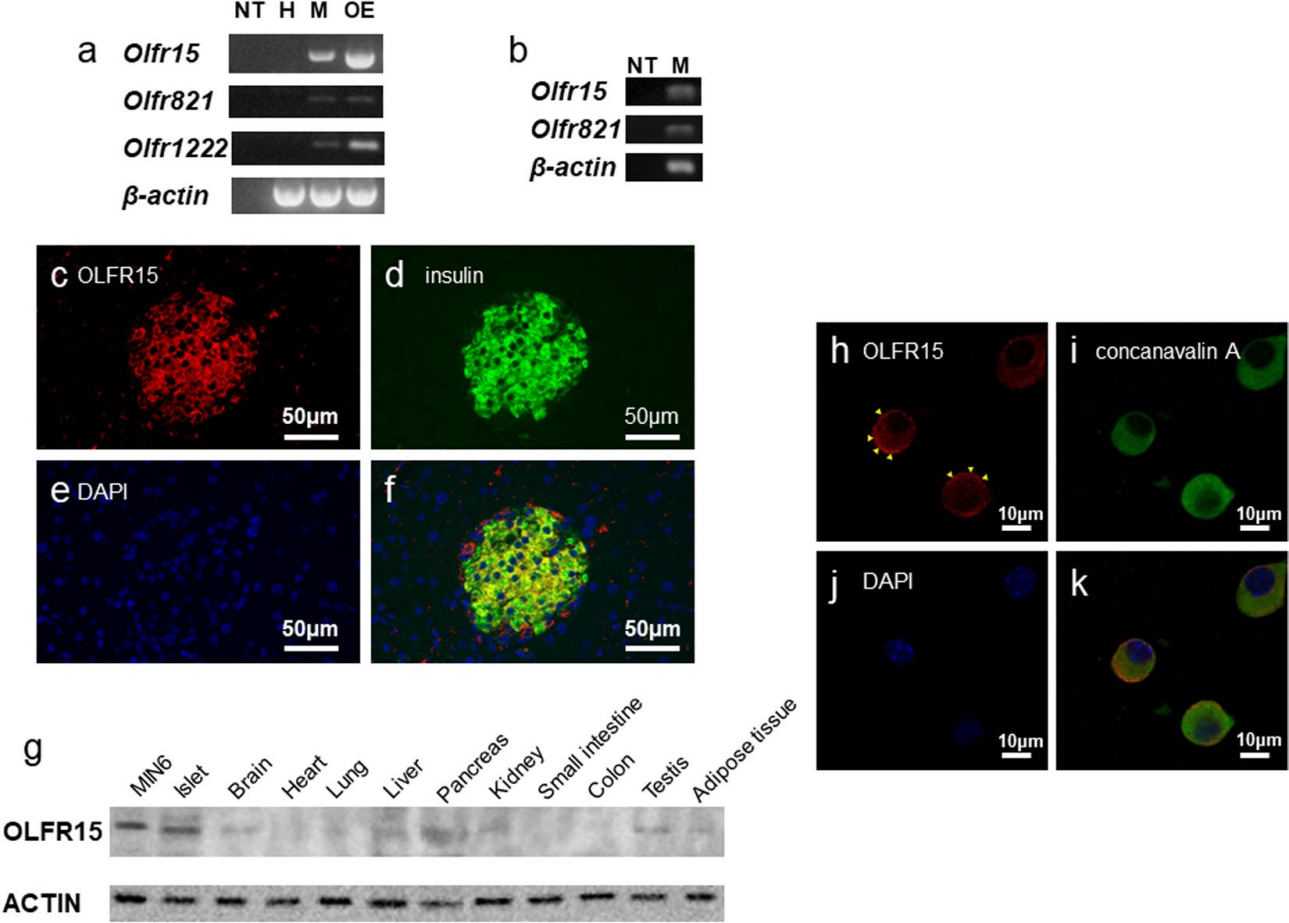
OLFR15 proteins are expressed on the plasma membranes of pancreatic β-cells. (**a**) mRNA expression levels of *Olfr15*, *Olfr821*, *Olfr1222* and *β-actin* in MIN6 cells and (**b**) those of *Olfr15*, *Olfr821*, and *β-actin* in a single MIN6 cell. NT, no template. H, Hepa 1-6. M, MIN6. OE, olfactory epithelia. Full-length gels are presented in Supplementary Fig. S10. (**c–f**) Immunofluorescence images of pancreatic islets or (**h–k**) confocal microscopic images of MIN6 cells, (**c,h**) demonstrating the expressions of OLFR15, (**d**) insulin, (**e,j**) DAPI, (**i**) concanavalin A, (**f**) coexpression of OLFR15, insulin and DAPI, and (**k**) coexpression of OLFR15, concanavalin A and DAPI. Arrowheads in (**h**) indicate sites of OLFR15 expression on the plasma membranes of MIN6 cells. concanavalin A, an ER marker protein. (**g**) The expressions of OLFR15 protein in MIN6 cells and murine islets, and the distribution of OLFR15 protein in murine tissues. Full-length blots are presented in Supplementary Fig. S10.

First, we performed microarray analyses using isolated pancreatic islets and a pancreatic β-cell line, MIN6, and, interestingly, obtained data suggesting that there are at least 47 ORs with mRNA expressions (Supplementary Table S1). Among them, we confirmed the expressions of *Olfr15*, *Olfr821* and *Olfr1222* in MIN6 cells by RT-PCR analysis (Fig. 1a). Furthermore, RT-PCR of individual MIN6 cells by single-cell RT-PCR revealed that mRNAs of *Olfr15* and *Olfr821* are simultaneously present in a single MIN6 cell (Fig. 1b), suggesting multi-OR expressions in individual MIN6 cells. We additionally performed immunohistochemical experiments on pancreatic tissues employing antibodies against OLFR15, OLFR821 and insulin. As shown in Fig. 1c–f and Supplementary Fig. S1a–d, both OLFR15 and OLFR821 proteins were detected in almost all β-cells as indicated by anti-insulin antibody staining, while no apparent signals of OLFR15 and OLFR821 were detected in exocrine cells. These results further support that OLFR15 and OLFR821 are simultaneously expressed in each pancreatic β-cell. The expression pattern of ORs observed in pancreatic β-cells is entirely different from that in olfactory epithelia in which each neuron expresses only one isoform of OR^5^. Among these ORs, we focused on OLFR15 in the present study, since OA, which enhances GSIS from the perfused rat pancreas^19^, reportedly interacts with OLFR15 over-expressed in 293 T cells^18^. We first examined OLFR15 expression level in various murine tissues. OLFR15 were expressed selectively in islets in mice, and expression level in islet cells were similar to that of MIN6 cells (Fig. 1g). Furthermore, confocal imaging revealed OLFR15 protein to be expressed mainly on the plasma membranes of MIN6 cells (Fig. 1h–k).

We then searched for genes homologous to *Olfr15* in two other species, humans and rats. Using the HomoloGene database at NCBI (http://www.ncbi.nlm.nih.gov/HomoloGene), we found the putative homologous rat and human genes to be *Olr1356* and *OR2C1*, respectively. The *Olfr15*, *Olr1356* and *OR2C1* genes all encode a 312-amino-acid polypeptide, and *Olr1356* and *OR2C1* have, respectively, 96% and 83% identities in their amino-acid sequences with *Olfr15* (Supplementary Fig. S2a). In addition, the expressions of *Olr1356* and *OR2C1* mRNA were clearly detected in rat and human pancreatic islets, respectively (Supplementary Fig. S2b).

### OLFR15 mediates amplification of GSIS by OA in MIN6 cells

**Figure 2:**
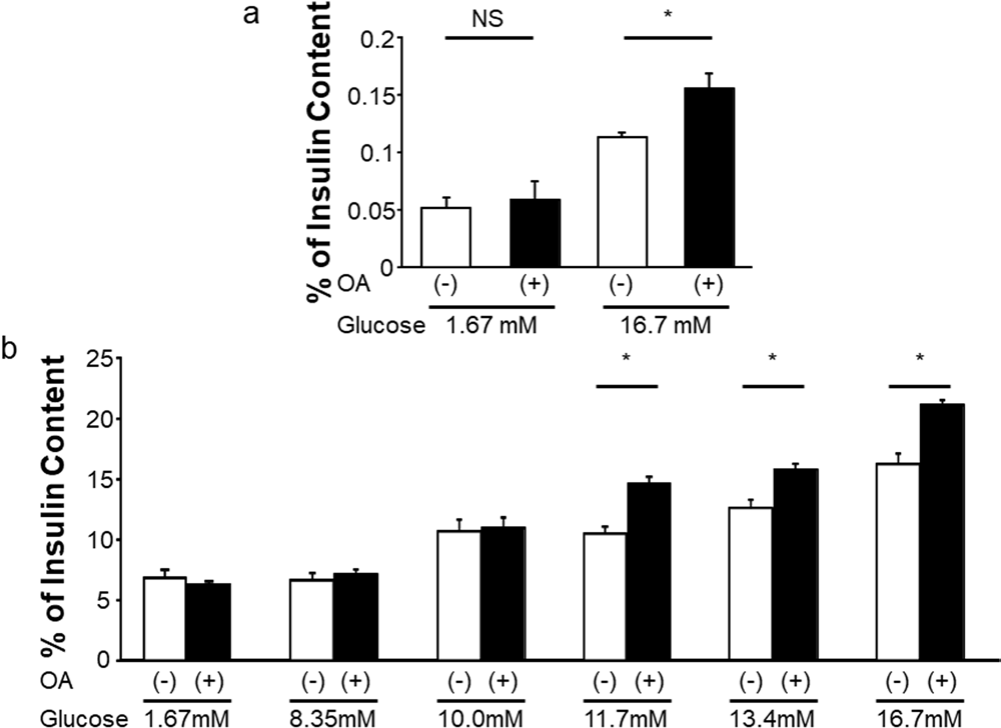
OA enhances GSIS from both isolated pancreatic islets and MIN6 cells. (**a**) Insulin secretion in murine islets at different glucose concentration with or without OA (n = 3 or 4 per group). (**b**) Insulin secretion at various glucose concentrations (1.67, 8.35, 10.0, 11.7, 13.4 and 16.7 mM) with or without OA in MIN6 cells (n = 4 to 6 per group). one-way ANOVA: *P < 0.05. NS, not significant. Data are presented as means ± SE.

OA, which is a MCFA contained in food and reportedly interacts with OLFR15^18^, was shown to enhance GSIS from the perfused rat pancreas^19^. However, it is unclear whether OA acts directly on pancreatic β-cells to enhance insulin secretion via OLFR15, or acts through other indirect pathways. Therefore, we observed effects of OA administration on insulin secretion using isolated murine pancreatic islets and MIN6 cells. In isolated murine pancreatic islets, adding OA significantly enhanced insulin secretion in response to 16.7 mM glucose, whereas insulin secretion did not change at 1.67 mM glucose (Fig. 2a). In addition, OA increased GSIS from isolated murine pancreatic islets in a dose-dependent manner (Supplementary Fig. S3). Furthermore, in MIN6 cells as well, adding OA to high concentrations of glucose (11.7, 13.4 and 16.7 mM) augmented insulin release, while these effects were absent when the glucose concentrations were lower than 10.0 mM (Fig. 2b). In contrast, OA did not significantly potentiate insulin secretion induced by 30 mM KCl (Supplementary Fig. S4). Collectively, these *ex vivo* and *in vitro* studies demonstrate that OA directly stimulates pancreatic β-cells, thereby promoting insulin secretion in response to high concentrations of glucose.

**Figure 3:**
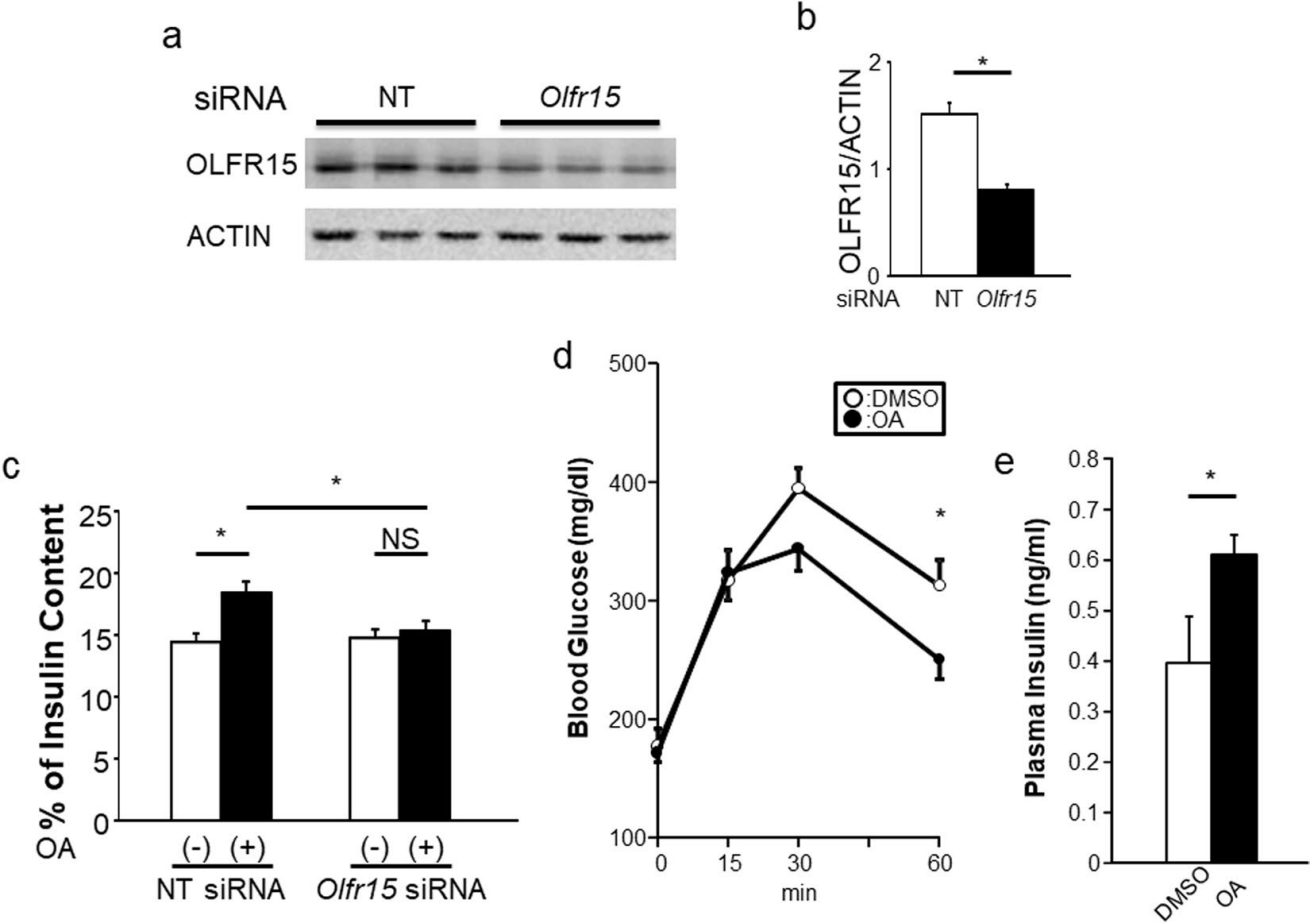
OA-induced enhancement of GSIS from MIN6 cells is abrogated by knockdown of OLFR15. (**a**) Protein expression levels of OLFR15 and ACTIN in MIN6 cells treated with NT siRNA or *Olfr15* siRNA (L-064657-01) for 72 hours. Full-length blots are presented in Supplementary Fig. S10. (**b**) Densitometric analyses for NT siRNA (n = 5) and *Olfr15* siRNA (L-064657-01) (n = 6) shown in (**a**). (**c**) MIN6 cells were transfected with NT siRNA or *Olfr15* siRNA (L-064657-01) for 72 hours, followed by monitoring for insulin secretion at 16.7 mM glucose with or without OA (n = 5 or 6 per group). Eight-week-old C57BL/6 J male mice were subjected to glucose tolerance tests performed in 30 minutes after an oral administration of dimethyl sulfoxide (DMSO) (n = 4) or OA (n = 5), followed by measurement of (**d**) blood glucose levels during the tests and (**e**) plasma insulin levels 15 minutes after glucose loading. NT, non-targeting. unpaired Student’s *t* test (**b** and **e**), one-way ANOVA (**c**) and two-way ANOVA (**d**): *P < 0.05. NS, not significant. Data are presented as means ± SE.

Although OA was reported to interact with OLFR15 in an artificial assay system^18^, it remains unclear whether OA is an functional ligand for OLFR15. Next, to examine whether OLFR15 is involved in the OA-enhanced GSIS from pancreatic β-cells, we knocked down OLFR15 expression in MIN6 cells using a specific siRNA. The specific siRNA for *Olfr15* significantly reduced the expression of OLFR15 protein in MIN6 cells (Fig. 3a,b). Consistently, immunocytochemical signals for OLFR15 in MIN6 cells were apparently decreased after siRNA treatment (Supplementary Fig. S5a,b). These findings also confirmed that the antibody used in this study selectively detects OLFR15. This OLFR15 knockdown blocked OA-induced enhancement of GSIS from MIN6 cells (Fig. 3c), while the OLFR15 knockdown itself did not affect GSIS (Supplementary Fig. S5c). In addition, another siRNA that knocks-down OLFR15 likewise suppressed GSIS enhancement induced by OA treatment (Supplementary Fig. S5d). These findings clearly indicate that OA is a functional ligand of OLFR15. Thus, OLFR15 is functionally expressed in MIN6 cells and mediates amplification of GSIS. Therefore, the ectopic OR system in pancreatic β-cells does actually affect insulin secretion.

### Peroral administration of the OLFR15 ligand potentiates insulin secretion after glucose loads *in vivo*

*Olfr15* and its homologs are likely to express in pancreatic β-cells in mice, rats and humans. Therefore, we next examined the effects of OLFR15 stimulation on insulin secretion and glucose metabolism *in vivo*. OA was administered orally at a dose (3 mM) at which oral fatty acid administration was reported to result in physiological and effective concentrations in the plasma^13^, followed by intraperitoneal glucose loading 30 min later. As shown in Fig. 3d,e and Supplementary Fig. S6a, OA administration significantly suppressed plasma glucose elevation with a significant increase in plasma insulin after glucose loading. In addition, OA enhanced GSIS in a dose-dependent manner *in vivo* (Supplementary Fig. S6b). Collectively, these findings suggest that OLFR15 stimulation delivered via a peroral approach enhances GSIS, leading to improvement of systemic glucose tolerance *in vivo*. Taken together with *ex vivo* and *in vitro* studies (Fig. 2a,b), OA-enhanced GSIS is likely to have a positive impact on maintaining glucose homeostasis after glucose loading.

### OLFR15-dependent GSIS enhancement is independent of the cyclic AMP (cAMP)-protein kinase A (PKA) pathway

**Figure 4:**
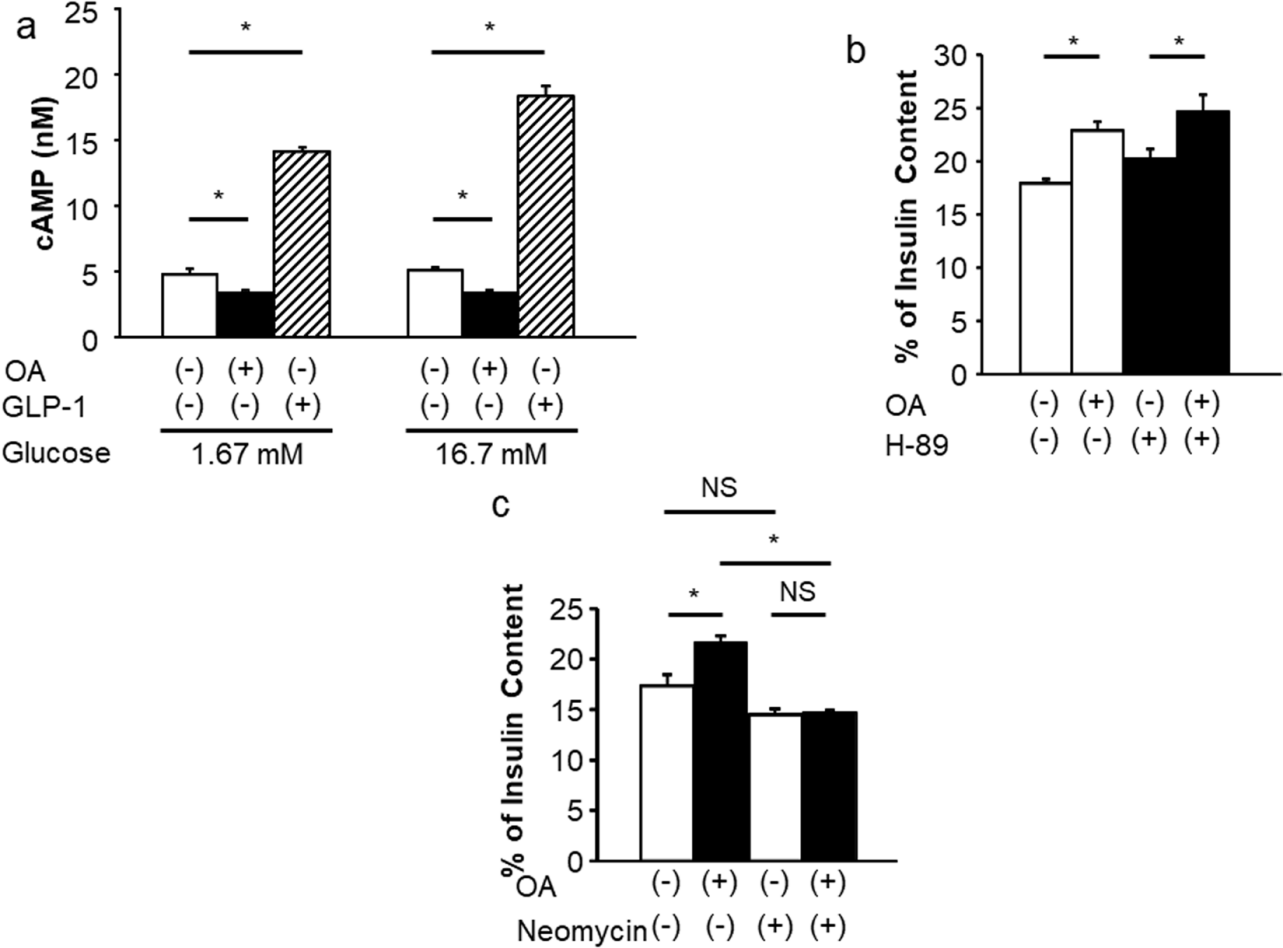
OLFR15-mediated GSIS enhancement is dependent on the PLC pathway but not the cAMP-PKA pathway. (**a**) cAMP levels in the absence or the presence of 0.5 mM OA or 10 nM GLP-1 with 1.67 mM and 16.7 mM glucose in MIN6 cells (n = 8 per group). (**b**) Insulin secretion at 16.7 mM glucose, with or without 0.5 mM OA, and with or without 1 μM H-89 in MIN6 cells (n = 5 or 6 per group). (**c**) Insulin secretion at 16.7 mM glucose, with or without 0.5 mM OA, and with or without 1.5 mM neomycin in MIN6 cells (n = 6 per group). one-way ANOVA: *P < 0.05. NS, not significant. Data are presented as means ± SE.

We next endeavored to identify which intracellular signals are involved in the OLFR15-dependent GSIS enhancement. In mammalian ORNs, stimulation of ORs reportedly raises the intracellular concentration of cAMP via adenylyl cyclase III (ACIII) through a mechanism mediated by Gα_olf_^20^. In addition, the cAMP-PKA pathway, which is activated by incretins or glucagon, is well known to enhance GSIS from pancreatic β-cells. Therefore, we examined whether the Gα_olf_-cAMP-PKA pathway is also involved in OA-induced enhancement of GSIS from MIN6 cells using two methods. First, we examined the effect of OA on intracellular cAMP concentrations in MIN6 cells. Contrary to our expectation, however, no increments in cAMP levels were observed in response to OA (Fig. 4a). Next, MIN6 cells were stimulated with OA in the absence or the presence of H-89, a selective PKA inhibitor^21^, followed by measurement of GSIS. H-89 did not inhibit GSIS enhancement with OA (Fig. 4b). Furthermore, *Gα*_*olf*_ mRNA was not detected in either isolated pancreatic islets or MIN6 cells, even using the RT-PCR procedure, while *Gα*_*olf*_ expression was clearly detected in olfactory epithelia (Supplementary Fig. S7a). Consistently, mRNA expressions *olfactory marker protein* (*Omp*), which acts as a modulator within the olfactory signal-transduction cascade in ORNs^22^, was undetectable in either MIN6 cells or pancreatic islets (Supplementary Fig. S7b). These results suggest a contribution of the Gα_olf_-cAMP-PKA pathway to OLFR15-dependent GSIS enhancement to be unlikely.

### PLC-β1 is involved in OLFR15-dependent GSIS enhancement

PLC activation was also reported to be involved in the enhancement of GSIS from pancreatic β-cells induced by the acetylcholine receptor and GPR40^17,23^. Therefore, we next examined involvement of the PLC pathway in OLFR15-mediated GSIS enhancement. MIN6 cells were stimulated with OA in the absence or the presence of neomycin, a PLC inhibitor^24^. GSIS enhancement in response to OA was almost completely abolished by neomycin treatment (Fig. 4c). In addition, we knocked down *Gnaq*, which is one of the Gq family proteins that reportedly activate PLC^25^, and obtained results showing that OA-enhanced GSIS was blunted by *Gnaq* knockdown (Supplementary Fig. S7c,d). Taken together, these results suggest that the Gq-PLC pathway, rather than the Gα_olf_-PLC pathway, is involved in OLFR15-dependent GSIS enhancement. To examine which isoform(s) of PLC is involved in OA-induced GSIS, we knocked down several PLC isoforms in MIN6 cells, followed by testing for the presence of GSIS enhancement in response to OA. We selected PLC isoforms reported to be expressed in pancreatic islets^26^ or a β-cell line^27^ and prepared specific siRNAs for each isoform. Specific siRNAs for *Plc-β1*, *-β3*, *-β4*, *-γ1* and *-δ4* significantly suppressed the expressions of the corresponding *Plc* isoforms in MIN6 cells (Supplementary Fig. S8a). In addition, knockdowns of PLC-β1 and PLC-γ1 were confirmed by immunoblotting (Supplementary Fig. S8b). Among these isoforms, only PLC-β1, when knocked down in MIN6 cells, modestly but significantly blunted the GSIS enhancement occurring in response to OA (Supplementary Fig. S9). Thus, these results suggest that PLC-β1 plays an important role in OLFR15-mediated GSIS enhancement, although we cannot rule out the possibility that another PLC isoform(s) is also involved.

### IP_3_ signaling plays an important role in OLFR15-dependent GSIS enhancement

**Figure 5:**
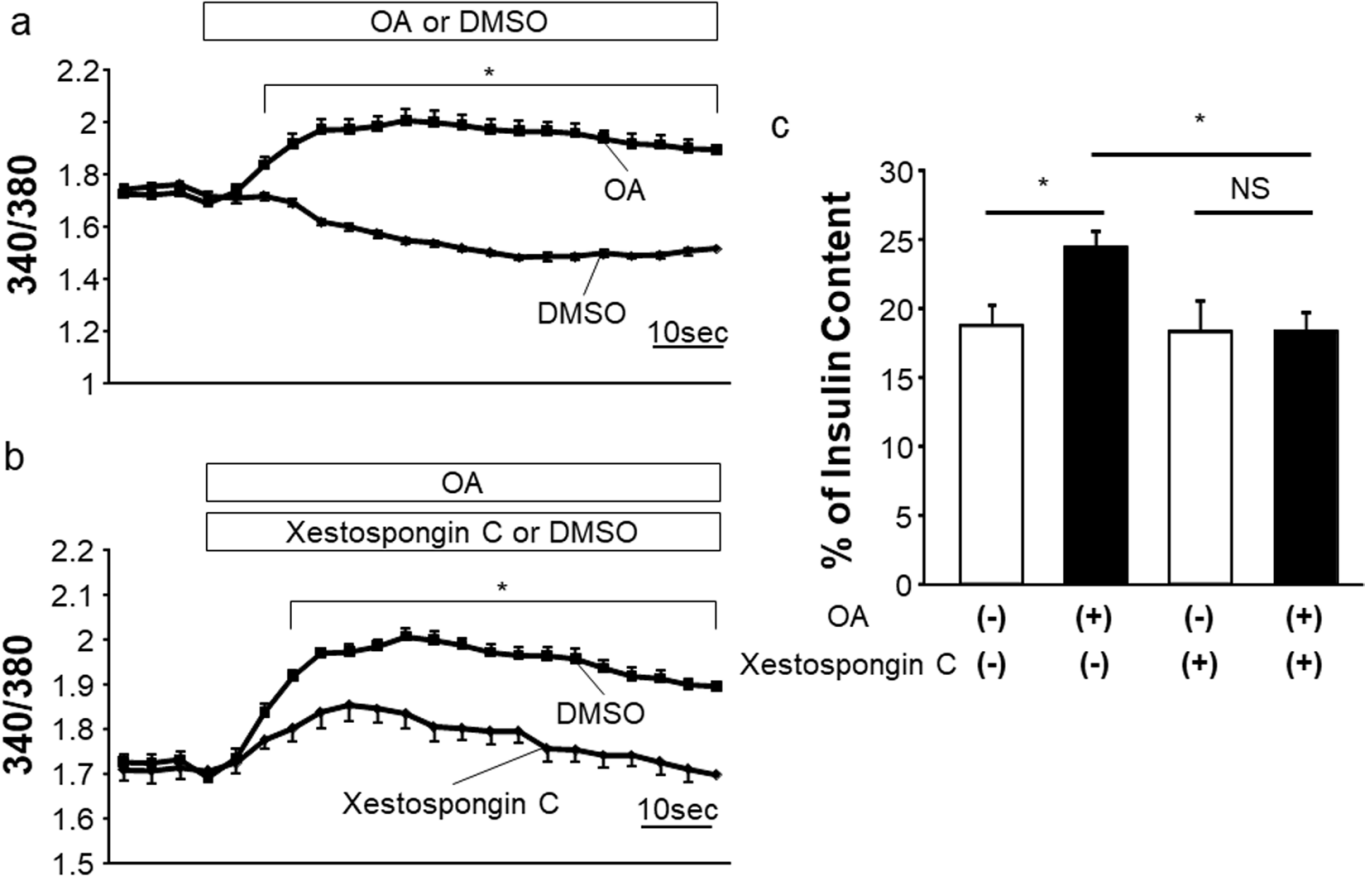
OLFR15-enhanced GSIS is abrogated by an IP_3_ receptor antagonist. (**a**) [Ca^2+^]i at 16.7 mM glucose with or without 0.5 mM OA in MIN6 cells (n = 8 per group). (**b**) [Ca^2+^]i at 16.7 mM glucose, with 0.5 mM OA, and with or without 2 μM Xestospongin C in MIN6 cells (n = 8 per group). (**c**) Insulin secretion at 16.7 mM glucose, with or without 0.5 mM OA, and with or without 2 μM Xestospongin C in MIN6 cells (n = 3 or 4 per group). one-way ANOVA (**c**) and two-way ANOVA (**a** and **b**): *P < 0.05. NS, not significant. Data are presented as means ± SE.

We further examined the pathway downstream from PLC, which is involved in GSIS enhancement. PLC activation reportedly leads to hydrolysis of phosphatidylinositol 4, 5-bisphosphate (PIP_2_), yielding diacylglycerol (DAG) and IP_3_, with the latter then binding to the IP_3_ receptor on the endoplasmic reticulum (ER) and subsequently inducing Ca^2+^ release from the ER. This induction of Ca^2+^ release reportedly increases insulin secretion^17,23^. Therefore, we first examined intracellular Ca^2+^ concentration ([Ca^2+^]i) alterations after OA treatment by measuring the fluorescence ratio of F340/F380 derived from Fura-2. As expected, [Ca^2+^]i was increased by OA treatment in MIN6 cells with a high glucose concentration (Fig. 5a), and this increase in [Ca^2+^]i was significantly suppressed by treatment with Xestospongin C, an IP_3_ receptor antagonist^21^ (Fig. 5b). Furthermore, Xestospongin C treatment blocked the GSIS enhancement induced by OA (Fig. 5c). These findings suggest that the IP_3_ receptor pathway plays a major role in OLFR15-enhanced GSIS.

Taken together, these observations indicate that the signaling pathway involved in OLFR15-mediated GSIS enhancement is not the Gα_olf_-cAMP-PKA pathway, but rather the PLC-β1- IP_3_-dependent pathway.

## Discussion

The olfactory system is known to function mainly in identifying foods, judging their edibility and detecting signals for a variety of social behaviors including the maintenance of territories^28^. ORs are G protein-coupled receptors (GPCRs) that mediate olfactory sensation as specialized chemo-sensors in ORNs^20^. OR expression is not, however, restricted to ORNs^7,8,29^. Since environmental substances obtained from foods also modulate insulin secretion, we speculated that there must be a chemo-sensing system(s), which recognizes the broad variation of available nutrients, in pancreatic β-cells. However, to the best of our knowledge, there are no reports indicating that either the ORs expressed in pancreatic β-cells or ectopic ORs in general have direct effects on insulin secretion. Herein, among ORs whose expressions were detected in pancreatic β-cells, we focused on OLFR15, since OA, which affects insulin secretion in the perfused rat pancreas^19^, reportedly interacts with OLFR15 in reconstituted 293 T cells^18^. We showed OA to promote GSIS in three different settings, i.e., isolated pancreatic islets *ex vivo*, the MIN6 pancreatic β-cell line *in vitro* and with peroral administration *in vivo*. In addition, knockdown of OLFR15 almost completely inhibited this OA-induced enhancement of GSIS (Fig. 3c). These results not only indicate that OA is a functional ligand of OLFR15 but also that OLFR15 mediates the insulin secretion-promoting effects of OA. The present results provide the first evidence indicating that an OR, OLFR15, is functionally expressed in pancreatic β-cells as a chemo-sensor, and promotes GSIS, thereby contributing to glucose homeostasis.

Incomplete knockdown (about 50%) of OLFR15 almost completely nullified GSIS enhancement. Similar complete inhibition of GPCR-mediated GSIS irrespective of incomplete GPCR knockdown was also reported previously^30^. In that prior paper, about 50% *Gpr40* knockdown completely blocked GSIS induced by long-chain fatty acid treatment in MIN6 cells. In addition, GPR40 signaling was reported to be mediated by the PLC-IP_3_ pathway^31^. As demonstrated in the present study, OLFR15 signaling is also mediated by the PLC-IP_3_ pathway. Taking our present and the previous reports together, when fatty acids function as ligands for GPCRs, there might be a mechanism that is responsible for the threshold in regard to receptor expression. Substantial GPCR levels may be needed to transduce the signals from fatty acids to the PLC-IP_3_ pathway, and to thereby enhance GSIS, although the precise mechanism remains unknown.

We analyzed mRNA expressions of pancreatic β-cells comprehensively by using microarray-based gene expression profiling and found as many as 47 ORs to be expressed in pancreatic β-cells. Gene expression results of several isoforms were confirmed by RT-PCR. Furthermore, we found OLFR15 and OLFR821 to be co-expressed in the same pancreatic β-cells. It is well known that the OR expression in ORN is strictly exclusive, i.e. each ORN harbors only one OR isoform. On the other hand, we demonstrated that each pancreatic β-cell expresses multiple OR isoforms simultaneously. Thus, the mechanism regulating OR expression in pancreatic β-cells is different from that in ORNs.

The OR family is one of the largest known mammalian gene families, with more than 1000 genes in mice^3,4^. Each OR has a wide range of ligands and each ligand may bind to several ORs with different affinities^18^. In addition, as described above, not only OLFR15 but also OLFR821, an orphan receptor, are simultaneously expressed in a pancreatic β-cell. These findings suggest that the OR system also enables pancreatic β-cells to recognize a wide variety of environmental substances included in foods. Thus, this system in pancreatic β-cells might play important roles in affecting insulin secretion in response to dietary substances, although intensive studies, including the use of mice deficient in all ORs in pancreatic β-cells, are needed to clarify the full picture of the physiological roles of the β-cell OR system.

In addition to pancreatic β-cells, immunohistochemical analysis revealed that OLFR15 protein was detectable in some non-β-cells (Fig. 1c–f). According to a previous report^32^, OLFR544 is expressed in pancreatic α-cells and regulates glucagon secretion. Therefore, OLFR15 might be involved in glucagon secretion.

As for the intracellular signal transduction in mammalian ORNs, Gα_olf_ is known to mainly mediate stimulation of the rapid synthesis of cAMP by ACIII^20^. In contrast, *Gα*_*olf*_ expression was not detected in either isolated pancreatic islets or MIN6 cells even by RT-PCR. Consistently, OA stimulation did not raise intracellular cAMP levels in MIN6 cells. In addition to the well-known cAMP-PKA pathway, odorants have been reported to activate the PLC pathway in mammalian ORNs^33,34^. In pancreatic β-cells, Gq-PLC activation reportedly leads to hydrolysis of PIP_2_ into DAG and IP_3_, which raises the cytoplasmic free Ca^2+^ concentration and subsequently enhances insulin secretion^17^. For example, PLC activation is reportedly involved in enhanced insulin secretion induced by cholecystokinin^35^ and interleukin-6^21^. In the present study, OA-enhanced GSIS was almost completely abolished by the PLC inhibitor neomycin (Fig. 4c) and was significantly diminished by suppression of PLC-β1 expression (Supplementary Fig. S9). Furthermore, both the increase in [Ca^2+^]i (Fig. 5b) and the subsequent GSIS (Fig. 5c) induced by OA were attenuated by an IP_3_ receptor antagonist. Collectively, these findings demonstrate that the PLC-β1-IP_3_-dependent pathway has an essential role in the OLFR15-mediated enhancement of insulin secretion. Incomplete inhibition of [Ca^2+^]i by Xestospongin C blocked nearly all OA-induced amplification of GSIS. Taken together with the observation that incomplete suppression of OLFR15 expression almost completely blocked OA-induced amplification of GSIS, these findings suggest a certain threshold in the signaling pathway for GSIS amplification. There are several examples indicating nutrient-induced GSIS in pancreatic β-cells to be dependent upon PLC activation; both GPR40, a receptor for long-chained FFAs^17^, and taste receptors^14,16^ expressed in pancreatic β-cells amplify GSIS via PLC activation. Thus, the PLC-IP_3_ signaling pathway may integrate information regarding the nutrients contained in foods and affect insulin secretion via several types of receptors.

In the present study, siRNA experiments revealed knockdown of PLC-β1, but not other isoforms, to significantly reduce OLFR15-enhanced GSIS. Since the degrees of the suppressed expression induced by each isoform-specific siRNA differed among isoforms (Supplementary Fig. S8a), these results do not exclude the possibility that another PLC isoform(s) contributes to OLFR15-enhanced GSIS. However, previous reports showed that PLC-β1 is activated by GPCRs^36^. In addition, we have reported that interleukin-6 enhances GSIS via PLC-β1 activation in MIN6^21^. Taken together, these observations indicate that PLC-β1 plays an important role in the mechanisms underlying GSIS enhancement including that mediated by OLFR15. The olfactory system in pancreatic β-cells may link intracellularly with the intrinsic machinery for augmentation of insulin secretion.

MCFAs, including OA, refer to a mixture of fatty acids which generally consist of 6-10 carbons. MCFAs are present at proportions of approximately 15%, 6.8%, 6.9%, 6.6% and 7.3% (of total fatty acid) in coconut oil, butter, milk, yogurt and cheese, respectively^37^. Medium-chain triglycerides (MCTs) are medium-chain fatty acid esters of glycerol. Dietary MCTs reportedly improve insulin secretion in patients with type 2 diabetes mellitus^38^. MCTs are rapidly hydrolyzed, resulting in absorption of MCFAs from the intestine^37^. Taking this finding together with the observation that the PLC-IP_3_ signaling pathway enhances GSIS in response to a number of nutrients, the ectopic OR system in pancreatic β-cells, as revealed in the present study, constitutes a potential therapeutic target for type 2 diabetes by enhancing insulin secretion.

In conclusion, we have demonstrated that OLFR15 is expressed in pancreatic β-cells and enhances GSIS through the PLC-IP_3_ pathway. This is the first report to present evidence that the ectopic OR system functionally exists in pancreatic β-cells and modulates whole-body glucose metabolism via regulating insulin secretion. The OR system in pancreatic β-cells may have a chemo-sensor function allowing recognition of environmental substances obtained from food, thereby contributing to maintenance of metabolic homeostasis. Thus, the OR system plays important roles in recognizing environmental factors, i.e. not only odorants from the air but also nutrients from food but the mechanisms, including receptor expressions (exclusive vs simultaneous), post-receptor signaling (cAMP vs IP_3_) and information integration (multi-cellular vs cell-autonomous) differ markedly between the nervous and endocrine systems. The OR system in pancreatic β-cells may have a chemo-sensor function allowing recognition of environmental substances obtained from food, thereby contributing to maintenance of metabolic homeostasis.

## Methods

### Materials

OA, neomycin (SIGMA, St. Louis, MO, USA), H-89 (Millipore, Billerica, MA, USA), GLP-1 (#22462, Anaspec, San Jose, CA, USA) and Xestospongin C (Enzo Life Sciences, Inc., Farmingdale, NY, USA) were commercially obtained. Rabbit antibodies to OLFR15 (NB110-75053, Novus Biologicals Inc., Littleton, CO, USA), OLFR821 (OSR00210W, Osenses, Australia), PLC-γ1 (#2822, Cell Signaling Technology, Beverly, MA, USA) and ACTIN (A2066, SIGMA), and murine antibody to PLC-β1 (sc-5291, Santa Cruz Biotechnology, Inc., Santa Cruz, CA, USA) were commercially obtained. Proteins derived from brain, heart, lung, liver, pancreas, kidney, small intestine, colon, testis and adipose tissue in C57BL/6 mice were purchased from Zyagen Laboratories (San Diego, CA, USA).

### Cell culture

The insulin-secreting β-cell line, MIN6-K8, which resembles native pancreatic islets in terms of the insulin secretion property^39,40^, were maintained in Dulbecco’s Modified Eagle Medium containing 25 mM glucose supplemented with 10% fetal calf serum.

### Microarray

An 8-week-old C57BL/6 J *db*/*db* mouse was purchased from the Jackson Laboratories. The mouse was given a standard laboratory diet (65% carbohydrate, 4% fat, 24% protein) and water *ad libitum* until euthanasia by cervical dislocation. The pancreas was removed immediately, embedded in OCT compound and flash-frozen. Samples were stored at − 80 °C until cryostat sectioning. Cryostat pancreatic sections (8 μm thick) were placed on PEN-coated slides (Leica Microsystems, Wetzlar, Germany) and stained with hematoxylin, allowing islets to be recognized. Immediately after staining, Laser micro-dissection (LMD) was carried out on a Leica AS LMD (Leica Microsystems)^41^. Total RNA was isolated from these pancreatic islets using the RNeasy Micro Kit (Qiagen, Valencia, CA, USA). At this point, total RNA from MIN6 cells was also obtained, using the RNeasy Micro Kit. Double-stranded cDNA was synthesized from 200 ng each of RNA from pancreatic islets and MIN6 cells. After cDNA synthesis, cRNA was synthesized using T7 RNA polymerase to incorporate Cyanine 3-CTP, according to the manufacturer’s instructions (Agilent Technologies, ON, Canada). The labeled cRNA was purified using an RNeasy Mini Kit (Qiagen, Valencia, CA, USA). RNA quality assessment was determined by Nanodrop (Thermo Scientific, MA, USA), Agilent 2100 Bioanalyzer and RNA 6000 NanoLab Chip Kit (Agilent Technologies, ON, Canada). Samples (1.65 μg each) were hybridized to Mouse Gene Expression 4 × 44 K v2 Microarray Kit (G4846A) at 65 °C for 17 hr. Slides were washed and then scanned by Agilent Microarray Scanner (G2565CA). Agilent Feature Extraction software version 10.7.3.1 was used to read the data.

### Single cell PCR

MIN6 cells were isolated in PBS containing 2 mM EDTA and sorted on the BD FACSAria II (BD Bioscience). Individual cells were sorted directly into 96 well PCR plates loaded with PCR buffer under single-cell mode. In total, 3 individual primer sets were pooled to a final concentration of 0.1 mM for each primer. Individual cells were sorted directly into 96 well PCR plates loaded with 9 μl of RT-PCR mix (5 μl CellsDirect reaction mix, Invitrogen; 1 μl primer pool; 0.2 μl SuperScript III RT platinum Taq Mix, Invitrogen; 2.8 μl nuclease free water) in each well. After centrifugation, the plates were immediately placed on a thermalcycler. Cell lysates and sequence-specific reverse transcription were performed at 50 °C for 15 minutes. Then, reverse transcriptase inactivation and Taq polymerase activation were achieved by heating at 95 °C for 2 minutes. Subsequently, cDNA went through 20 cycles of sequence-specific amplification by denaturing at 95 °C for 15 seconds, annealing, and elongation at 60 °C for 4 minutes in the same tube. After preamplification, the preamplified products were treated with Exonuclease I (New England Biolabs). These products were then diluted 5-fold prior to analysis. Amplified single-cell samples were analyzed using RT-PCR^42^.

### Studies with isolated islets and MIN6 cells

Pancreatic islets were isolated from 8- to 9-week-old C57BL/6 J mice and 10- to 11-week-old SD rats by retrograde injection of collagenase (SIGMA) into the pancreatic duct according to the standard procedure, as described previously^43,44^. Isolated islets were maintained in RPMI640 medium containing 11.1 mM glucose. For insulin secretion studies, batches of 10 islets or MIN6 cells were washed with modified Krebs–Ringer bicarbonate buffer (KRBB) [135 mM NaCl, 3.6 mM KCl, 0.5 mM NaH_2_PO_4_, 0.5 mM MgCl_2_, 1.5 mM CaCl_2_, 2 mM NaHCO_3_, 10 mM HEPES and 0.1% BSA]. After a 30-min pre-incubation in KRBB containing 1.67 mM glucose, islets or MIN6 cells were treated for 60 minutes in KRBB supplemented with either 1.67 or 16.7 mM glucose in the absence or the presence of 0.5 mM OA. Insulin contents of isolated islets or MIN6 cells were measured after acid ethanol (1.5% HCL in 75% ethanol) extraction.

Human pancreatic islet cDNAs were commercially obtained from Primary Cell Co. Ltd. (Sapporo, Japan).

### Small interfering RNA transfection

The following siRNA oligonucleotides were purchased from Thermo Fisher Scientific (Waltham, MA, USA); *Olfr15* (ON-TARGETplus SMART pool: L-064657-01), *Olfr15*(siGENOME: D-064657-03 and siGENOME: D-064657-04), *Plc-β1* (ON-TARGETplus SMART pool: L-062031-01), *Plc -β3* (ON-TARGETplus SMART pool: L-040969-01), *Plc -β4* (ON-TARGETplus SMART pool: L-041184-01), *Plc -γ1* (ON-TARGETplus SMART pool: L-040978-01), *Plc -δ4* (ON-TARGETplus SMART pool: L-040349-01) and *Gnaq* (ON-TARGETplus SMART pool: L-040927-00). When D-064657-03 and D-064657-04 were used, they were mixed together, according to the manufacturer’s protocol. MIN6 cells were transfected with siRNAs (100 nM) using DharmaFECT 1 or 3 Transfection Reagent (Thermo Fisher Scientific). These cells were analyzed 48 hours after transfection, with the exception of *Olfr15*. MIN6 cells transfected with *Olfr15* siRNA were analyzed 72 hours after transfection.

### Evaluation of gene expression by RT-PCR and real-time RT-PCR

RNA was isolated from isolated olfactory epithelium, isolated islets, and MIN6 cells using the RNeasy Mini Kit or the RNeasy Micro Kit (Qiagen, Valencia, CA, USA). RNA was reverse transcribed into cDNAs using a QuantiTect Reverse Transcription Kit (Qiagen). *Olfr15*, *Olfr821*, *Olfr1222*, *Olr1356*, *OR2C1*, rat and human *β-actin*, *Gα*_*olf*_, *Omp* and murine *β-actin*, were detected by RT-PCR, with the oligonucleotides presented in Supplementary Table S2.

On the other hand, template cDNAs were evaluated with a real-time PCR quantitative system (Light Cycler Quick System 350 S; Roche Diagnostics, Mannheim, Germany), with the oligonucleotides presented in Supplementary Table S3. The relative amount of mRNA was calculated with *β-actin* mRNA as the invariant control.

### Animals

Male C57BL/6 J mice were purchased from Charles River Japan (Kanagawa, Japan) and SLC (Shizuoka, Japan). Male Sprague-Dawley (SD) rats were purchased from Japan SLC (Hamamatsu, Japan). Mice and rats were housed in an air-conditioned environment, with a 12-h light-dark cycle (light on at 09:00 A.M.), and fed a regular unrestricted diet. All animal studies were conducted in accordance with Tohoku University institutional guidelines. The experimental protocols were approved by the Institute for Animal Experimentation in Tohoku University Graduate School of Medicine (Permit Number: 2017-067).

### Western blotting

Tissue homogenates and cell lysates were subjected to SDS-PAGE and probed with primary antibodies. The specific bands were subjected to densitometry analysis using Image J software (http://rsbweb.nih.gov/ij/index.html).

### Immunohistochemical detection of OLFR15 and OLFR821 protein

For OLFR15, OLFR821 and insulin staining, murine pancreases were fixed with 10% formalin and embedded in paraffin. Tissue sections were cut at a thickness of 4 μm, de-paraffinized, re-hydrated and rinsed in 0.01 M PBS.

Sections for OLFR15 and insulin co-staining were microwaved for 20 minutes and incubated with 10% goat serum for 30 minutes at room temperature. The sections were then incubated overnight at 4 °C with OLFR15 antibody at a dilution of 1:100. After washing, the sections were incubated with Alexa 546 goat anti-rabbit IgG (A11010, Invitrogen) (1:500) for 60 minutes at room temperature. Next, these sections were incubated with 10% goat serum for 30 minutes at room temperature. The sections were then incubated with insulin antibody (I2018, SIGMA) at a dilution of 1:2000 for 60 minutes at room temperature. The sections were then incubated with Alexa 488 goat anti-mouse IgG (A21121, Invitrogen) (1:500) for 60 minutes at room temperature.

Sections for OLFR821 and insulin co-staining were digested employing a protease (415231, Nichirei Bioscience, Tokyo, Japan). After washing, the sections were incubated with 10% goat serum for 30 minutes at room temperature. The sections were incubated overnight at 4 °C with OLFR821 antibody at a dilution of 1:500. After washing, the sections were incubated with rabbit-specific biotinylated antibody (426012, Nichirei Bioscience) for 30 minutes at room temperature, followed by alkaline phosphatase-conjugated streptavidin (s11225, Invitrogen) (1:100) for 45 minutes at room temperature. Next, these sections were incubated with 10% goat serum for 30 minutes at room temperature. The sections were then incubated with insulin antibody (I2018, SIGMA) at a dilution of 1:2000 for 30 minutes at room temperature. The sections were then incubated with Alexa 488 goat anti-mouse IgG (A21121, Invitrogen) (1:500) for 30 minutes at room temperature. The sections were subsequently mounted and covered using a mounting medium with DAPI (VECTASHIELD; Vector Laboratories).

### Immunofluorescence microscopy

MIN6 cells were fixed with 4% formaldehyde at room temperature for 10 minutes, then washed with PBS and permeabilized with PBS/0.1% Triton-X-100 for another 8 minutes at room temperature. Cells were incubated with anti-OLFR15 antibody (1:500 dilution), and then secondary antibody [Alexa 546 goat anti-rabbit IgG (1:500)] was used to detect the primary antibody. The ERs were stained on fixed cells with concanavalin A/Alexa 488(C11252, Invitrogen). The cell nuclei were counterstained with DAPI (H-1200, Vector LaboratoriesInc., Burlingame, CA, USA). Cells were visualized and photographed with a Leica TCS SP8 STED confocal image collection apparatus, using a 40 × oil immersion lens.

### Image quantification

After siRNA treatment, MIN6 cells were fixed in 4% paraformaldehyde, and labeled with antibodies against OLFR15 as described above. From each sample these images were taken by means of fluorescence microscopy (Keyence Biorevo BZ-9000). As previously reported, each cell was densitometrically analyzed employing Keyence BZ-9000 Analyzer software^45^. Values obtained from control MIN6 cells were set to 100%.

### Glucose tolerance tests

Glucose tolerance tests were performed on fasted (9 h, daytime) mice. The 8-week-old C57BL/6 J mice were given glucose (1 g/kg of body weight) intraperitoneally, followed by measurement of blood glucose and insulin levels^46^. OA was administered perorally at a dose calculated to yield the desired final plasma concentration (3 mM) 30 minutes before intraperitoneal glucose administration.

### Blood analysis

Blood glucose was assayed with Glutestmint (Sanwa Kagaku Kenkyusho, Nagoya, Japan). ELISA kits were used to measure plasma insulin (Morinaga Seikagaku Kenkyusho, Yokohama, Japan). The amount of insulin secretion was normalized by cellular insulin content in both *in vitro* and *ex vivo* studies.

### cAMP assay

MIN6 cells were incubated for 30 minutes in the absence or the presence of 0.5 mM OA with either 1.67 or 16.7 mM glucose. Cellular cAMP levels were determined using a cAMP dynamic 2 kit (Cisbio Bioassays, Codolet, France).

### Measurements of [Ca^2+^]i

[Ca^2+^]i levels were determined using a Calcium kit II-Fura 2 (Dojindo Laboratories, Kumamoto, Japan). Measurements of the fluorescence ratio of F340/F380, reflecting [Ca^2+^]i, were demonstrated by Flexstation 3 (Molecular Devices).

### Statistical analysis

The statistical significance of differences between groups was assessed employing the unpaired Student’s *t* test, one-way ANOVA or two-way ANOVA followed by Fisher’s post-hoc test, as appropriate. Data are presented as means ± standard error (SE). A value of *P* < 0.05 was considered to indicate a statistically significant difference.

## Electronic supplementary material

Supplementary Information
