# Supplementary material for: Olfactory receptors are expressed in pancreatic β-cells and promote glucose-stimulated insulin secretion
Source: Sci Rep. 2018 Jan 24;8:1499. doi: 10.1038/s41598-018-19765-5 (PMC5784078; doi:10.1038/s41598-018-19765-5)
Supplement: Supplementary file 1 — Supplementary Information [file 41598_2018_19765_MOESM1_ESM.pdf]

Supplementary Information:

Supplementary Figures S1-S10
Supplementary Tables S1-S3

Olfactory receptors are expressed in pancreatic β-cells and promote glucose-stimulated insulin secretion

Yuichiro Munakata, Tetsuya Yamada, Junta Imai, Kei Takahashi, Sohei Tsukita, Yuta Shirai, Shinjiro Kodama, Yoichiro Asai, Takashi Sugisawa, Yumiko Chiba, Keizo Kaneko, Kenji Uno, Shojiro Sawada, Hiroyasu Hatakeyama, Makoto Kanzaki, Jun-ichi Miyazaki, Yoshitomo Oka and Hideki Katagiri

Supplementary Figure S1: The expressions of OLFR821 protein are detectable in murine pancreatic islets.

(**a-d**) Immunofluorescent images of pancreatic islets demonstrating the expressions of (**a**) OLFR821, (**b**) insulin, (**c**) DAPI, and (**d**) coexpression of OLFR821, insulin and DAPI.

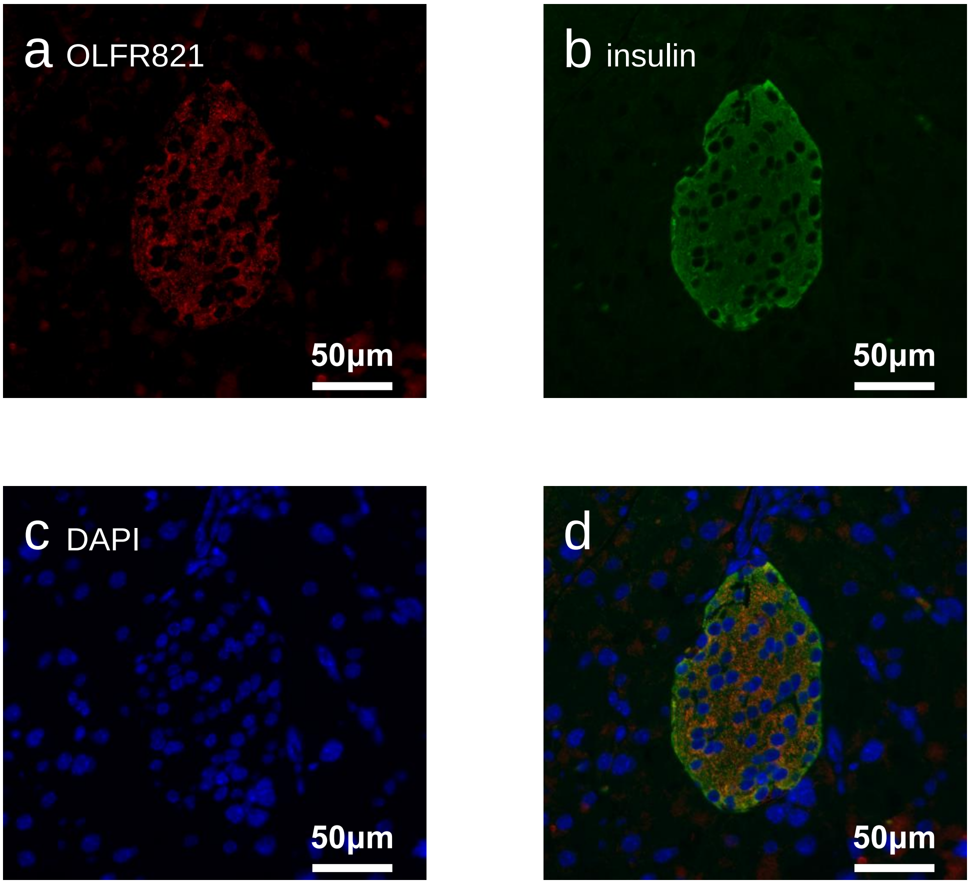

Supplementary Figure S2: The expressions of *Olr1356* and *OR2C1* mRNA are detectable in rat and human pancreatic islets, respectively.

(**a**) Amino acid sequence alignment of *Olfr15* from mouse, *Olr1356* from rat and *OR2C1* from human islets. The different sequences from *Olfr15* are underlined. (**b**) mRNA expression levels of *Olr1356* and β-actin in rat islets, and those of *OR2C1* and *β-actin* in human islets.

a

```
Olfr15 (mouse)   MEVDSNSSSGSFILMGVSDHPHLEIIFFAVILASYLLTLVGNLTIILLSRLDAR
Olr1356(Rat)     MEVGSNISSGSFILMGISNHPQLEIIFFVVILSSYLLTLVGNLTIILLSRLDAR
OR2C1  (Human)   MDGVNDSSLQGFVLMGISDHPQLEMIFFIAILFSYLLTLLGNSTIILLSRLEAR

Olfr15 (mouse)   LHTPMYFFLSNLSSLDLAFTTSSVPQMLKNLWGPDKTISYGGCVTQLYVFLWLG
Olr1356(Rat)     LHTPMYFFLSNLSSLDLAFTTSSVPQMLKNLWGPDKTISYGGCVTQLYVFLWLG
OR2C1  (Human)   LHTPMYFFLSNLSSLDLAFATSSVPQMLINLWGPGKTISYGGCITQLYVFLWLG

Olfr15 (mouse)   ATECILLVVMAFDRYVAVCRPLHYMTVMNPRLCWGLAAISWLGGLGNSVIQSTF
Olr1356(Rat)     ATECILLVVMAFDRYVAVCRPLHYMTVMNPRLCWVLAAISWLGGLGNSVIQSTF
OR2C1  (Human)   ATECILLVVMAFDRYVAVCRPLRYTAIMNPQLCWLLAVIACLGGLGNSVIQSTF

Olfr15 (mouse)   TLQLPFCGHRKVDNFLCEVPAMIKLACGDTSLNEAVLNGVCTFFTVVPVSVILV
Olr1356(Rat)     TLQLPFCGHRKVDNFLCEVPAMIKLACGDTSLNEAVLNGVCTFFTAVPLSIILV
OR2C1  (Human)   TLQLPLCGHRRVEGFLCEVPAMIKLACGDTSLNQAVLNGVCTFFTAVPLSIIVI

Olfr15 (mouse)   SYCFIAQAVMKIRSVEGRRKAFNTCVSHLVVVFLFYGSAIYGYLLPAKSSNQSQ
Olr1356(Rat)     SYCFIAQAVMKIRSVEGRRKAFNTCVSHLVVVFLFYGSAIYGYLLPAKSSNQDQ
OR2C1  (Human)   SYCLIAQAVLKIRSAEGRRKAFNTCLSHLLVVFLFYGSASYGYLLPAKNSKQDQ

Olfr15 (mouse)   GKFISLFYSVVTPMVNPLIYTLRNKEVKGALGRLLGKGRGAS
Olr1356(Rat)     GKFISLFYSVVTPMVNPLIYTLRNKEVKGALGRLLGKGRGAS
OR2C1  (Human)   GKFISLFYSLVTPMVNPLIYTLRNMEVKGALRRLLGKGREVG
```

b

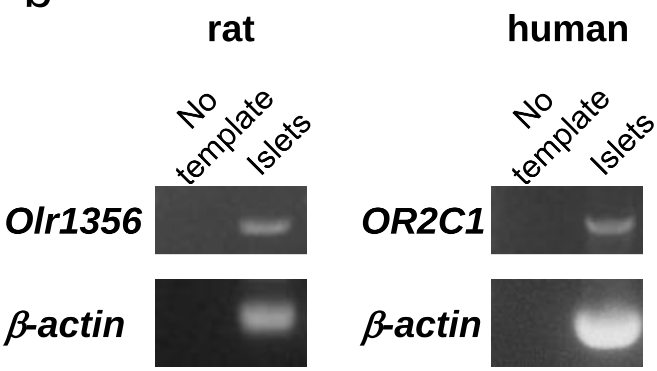

Munakata et al Supplementary Figure S3

Supplementary Figure S3: OA enhanced GSIS from murine islets in a dose-dependent manner. Insulin secretion was measured in murine islets incubated for 60 minutes in 16.7 mM glucose supplemented with KRBB and OA (either 0.1, 0.5 or 1.0 mM; n = 3 or 4 per group). one-way ANOVA: **P* < 0.05. Data are presented as means ± SE.

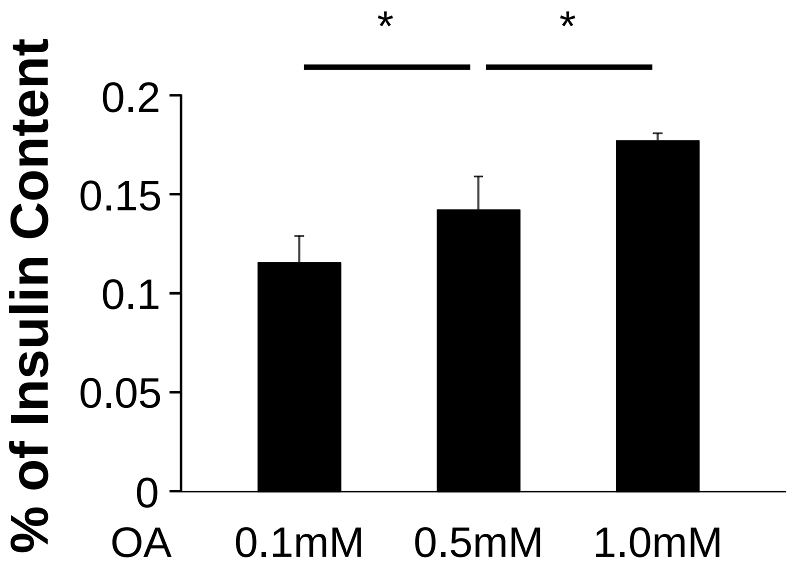

Supplementary Figure S4: OA-enhanced GSIS is not affected by KCl treatment in MIN6 cells. Insulin secretion was measured in MIN6 cells incubated for 60 minutes in 1.67 mM glucose supplemented KRBB, with or without 0.5 mM OA, and with or without 30 mM KCl (n = 4 to 6 per group). one-way ANOVA: NS, not significant. Data are presented as means ± SE.

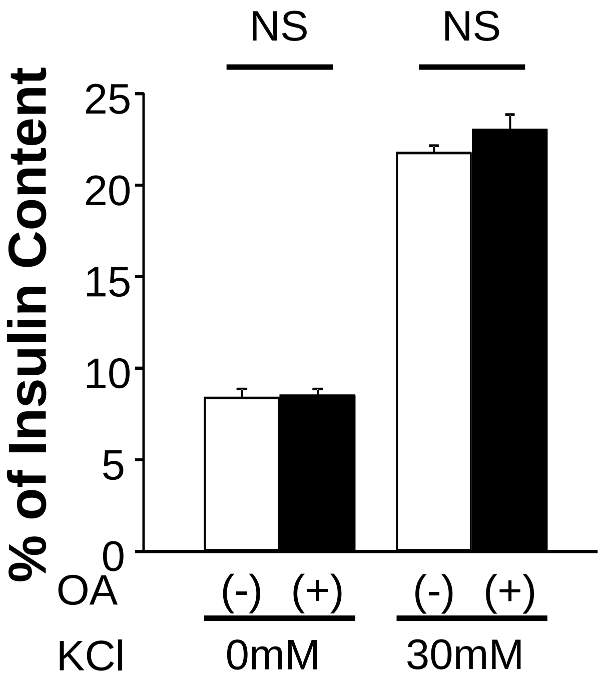

Munakata et al Supplementary Figure S5

Supplementary Figure S5: *Olfr15* siRNA treatments suppress the protein expression of OLFR15 in MIN6 cells.

(**a**) Immunofluorescence images for *Olfr15* in MIN6 cells treated with NT or *Olfr15* siRNA (L-064657-01) for 72 hours. (**b**) OLFR15 protein expression levels in MIN6 cells treated with NT siRNA (n=49) or *Olfr15* siRNA (L-064657-01) (n=34) for 72 hours, as revealed by quantitative immunocytochemistry. (**c**) MIN6 cells were transfected with NT siRNA or *Olfr15* siRNA (L-064657-01) 72 hours prior to measurement of insulin secretion at 1.67mM or 16.7 mM glucose (n = 6 per group). (**d**) MIN6 cells were transfected with NT siRNA or another *Olfr15* siRNA (mixture of D-064657-03 and D-064657-04) 72 hours prior to measurement of insulin secretion at 16.7 mM glucose alone or with OA (n = 5 or 6 per group). NT, non-targeting. unpaired Student's *t* test (**b, d**) and one-way ANOVA (**c**) : **P* < 0.05. NS, not significant. Data are presented as means ± SE.

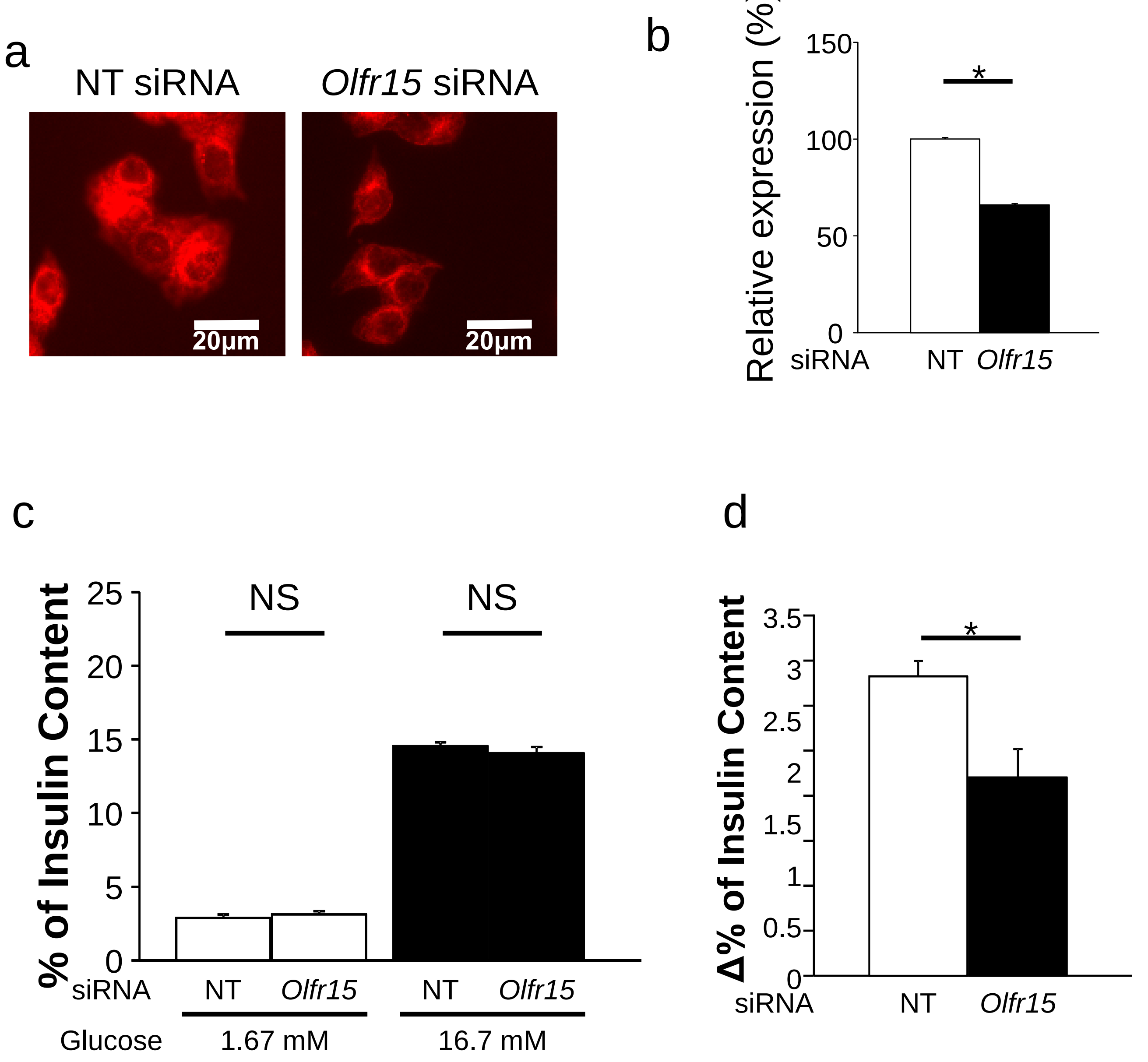

Munakata et al Supplementary Figure S6

Supplementary Figure S6: OA enhances GSIS *in vivo*.

(**a**) Eight-week-old C57BL/6J male mice were subjected to oral administration of dimethyl sulfoxide (DMSO) or 3 mM OA, followed by measurement of plasma insulin 30 minutes later. (**b**) Eight-week-old C57BL/6J male mice were subjected to oral administration of OA (3, 30 or 50 mM), followed by intraperitoneal glucose tolerance test 30 minutes later. Plasma insulin levels were measured 15 minutes after glucose loading. NT, non-targeting. unpaired Student's *t* test (**a**) and one-way ANOVA (**b**) **:** **P* < 0.05. NS, not significant. Data are presented as means ± SE.

a

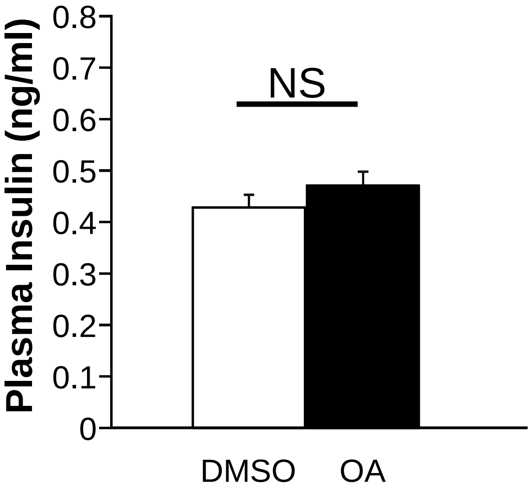

b

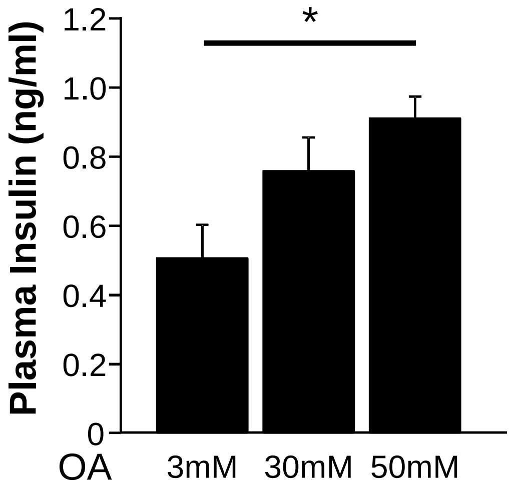

Munakata et al Supplementary Figure S7

Supplementary Figure S7: OLFR15-mediated GSIS enhancement depends on Gnaq pathway rather than $G\alpha_{olf}$ pathway.

(**a**) mRNA expression levels of $G\alpha_{olf}$ and *β-actin* in MIN6 cells, murine islets and olfactory epithelia. (**b**) mRNA expression levels of *Omp* and *β-actin* in MIN6 cells, murine islets and olfactory epithelia. (**c**) NT siRNA (open bars; n = 3) or siRNA specific for *Gnaq* (closed bar; n = 3) was applied to MIN6 cells. After a 48-hour incubation, the cells were subjected to real-time RT-PCR analysis. *Gnaq* expression levels in MIN6 cells were quantified and normalized relative to *β-actin* mRNA levels. (**d**) MIN6 cells were transfected with NT siRNA or the siRNA specific for *Gnaq* 48 hours prior to measurement of insulin secretion at 16.7 mM glucose alone or with OA (n = 4 or 6 per group). NT, non-targeting. unpaired Student's *t* test (**c**) and one-way ANOVA (**d**) : *$P < 0.05$. NS, not significant. Data are presented as means ± SE.

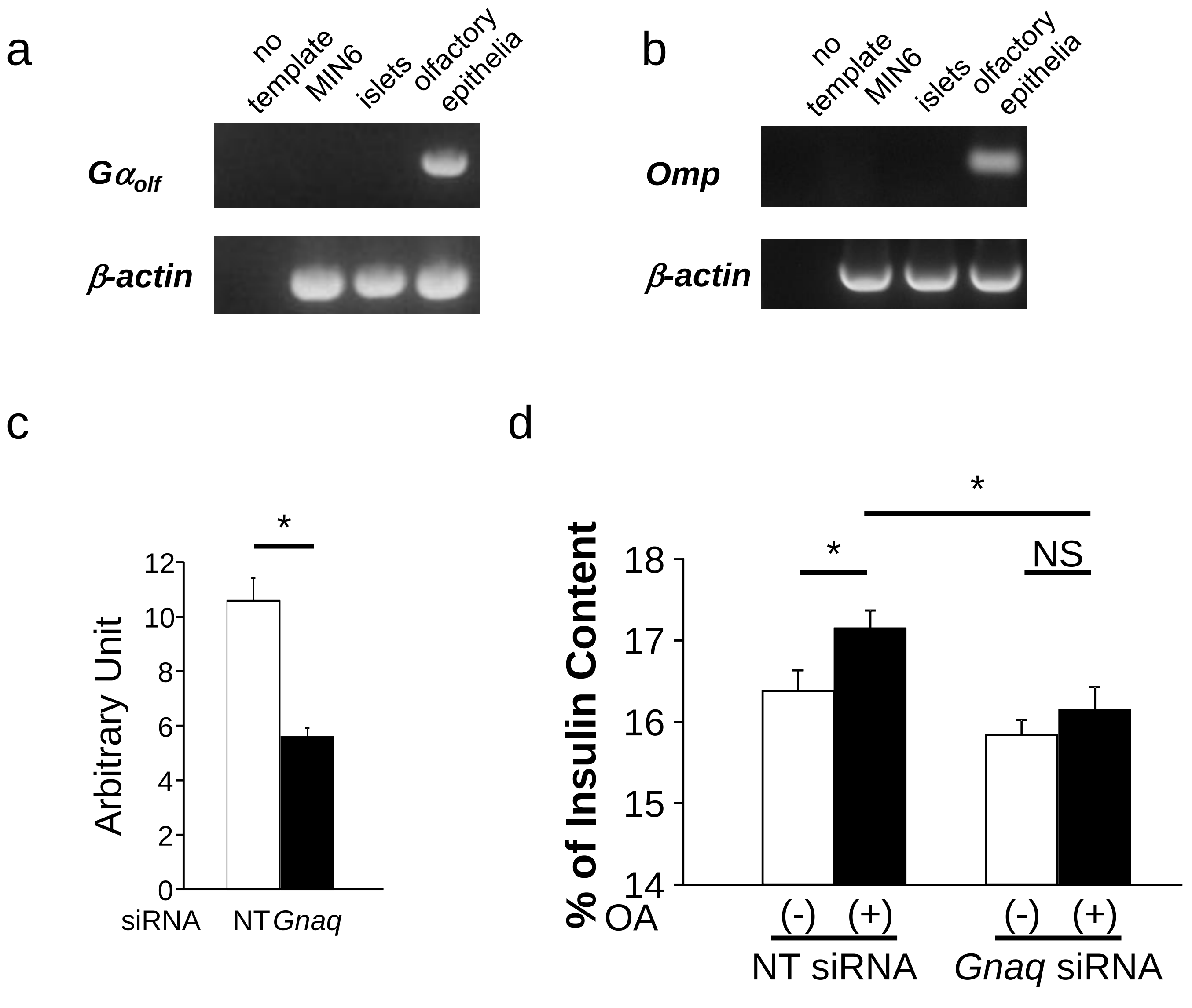

Munakata et al Supplementary Figure S8

Supplementary Figure S8: Specific siRNA treatments in MIN6 cells suppress the mRNA expression of each *Plc* isoform.

(**a**) NT siRNA (open bars; n = 3) or siRNA specific for each *Plc* isoform (closed bar; n = 3) was applied to MIN6 cells. After a 48-hour incubation, the cells were subjected to real-time RT-PCR analysis. *Plc* expression levels in MIN6 cells were quantified and normalized relative to *β-actin* mRNA levels. (**b**) Protein expression levels of PLC-β1, PLC-γ1 and actin in MIN6 cells treated with NT siRNA or *Plc* isoform siRNA for 48 hours. NT, non-targeting. unpaired Student's *t* test: *$P < 0.05$. Data are presented as means ± SE.

a

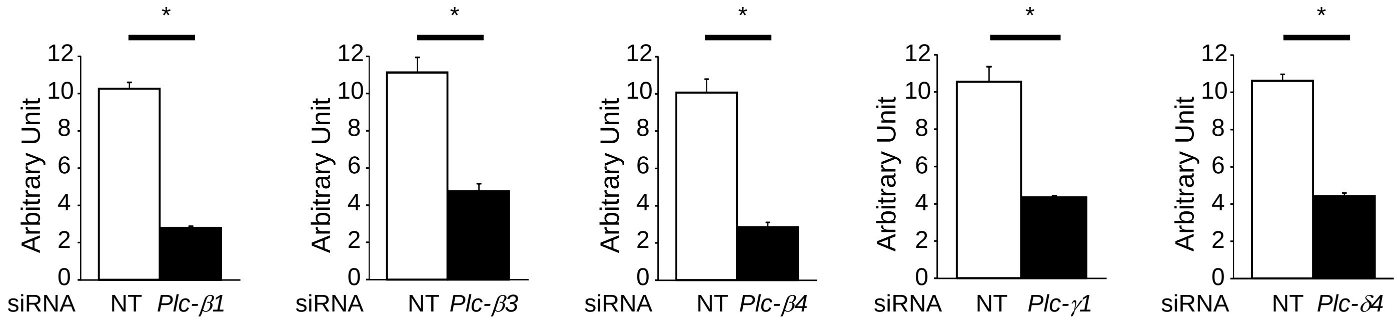

b

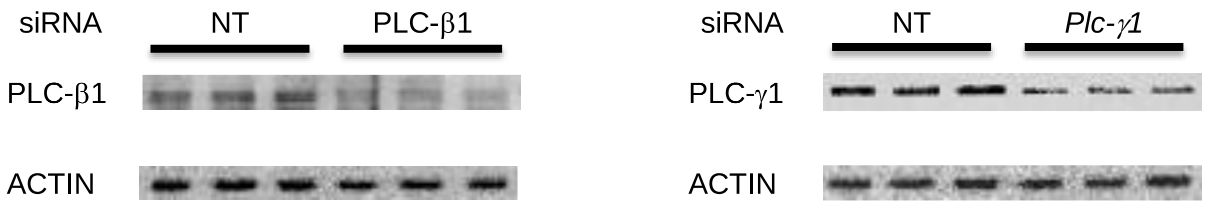

Munakata et al Supplementary Figure S9

Supplementary Figure S9: PLC-β1 is involved in Olfr15-enhanced GSIS.

MIN6 cells were transfected with NT siRNA or siRNA specific for each *Plc* isoform for 48 hours, followed by monitoring for insulin secretion at 16.7 mM glucose with or without OA (n = 6 per group). NT, non-targeting. one-way ANOVA: *$P < 0.05$. NS, not significant. Data are presented as means ± SE.

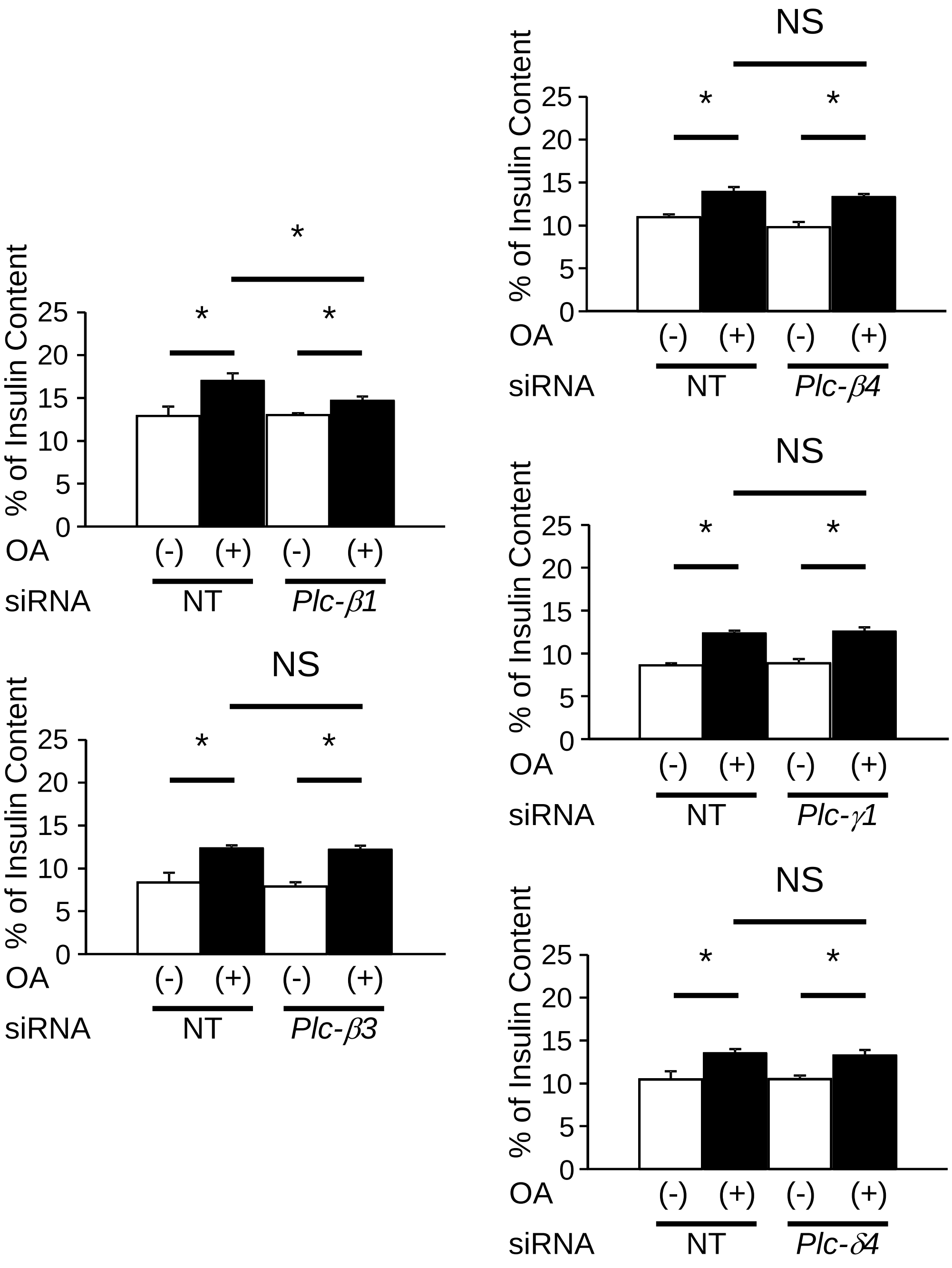

Munakata et al Supplementary Figure S10

Supplementary Figure S10: Uncropped images of key panels in main figures.
Red boxes indicate the cropped portion of each immunoblot presented in the corresponding main figures.

Figure 1a

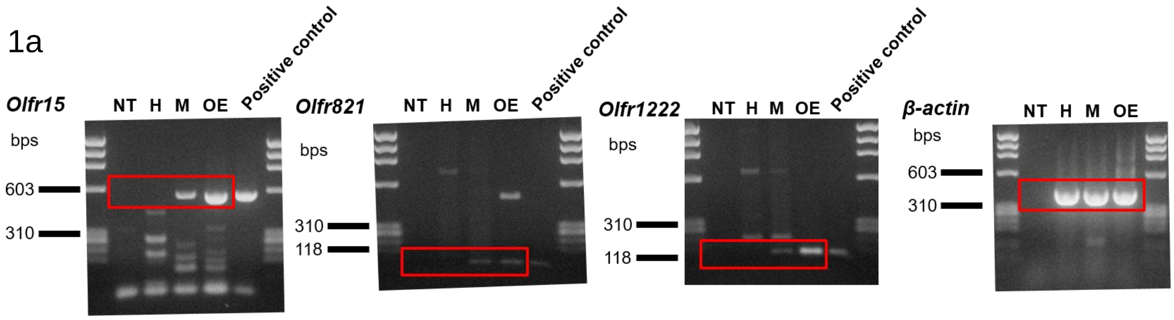

Figure 1b

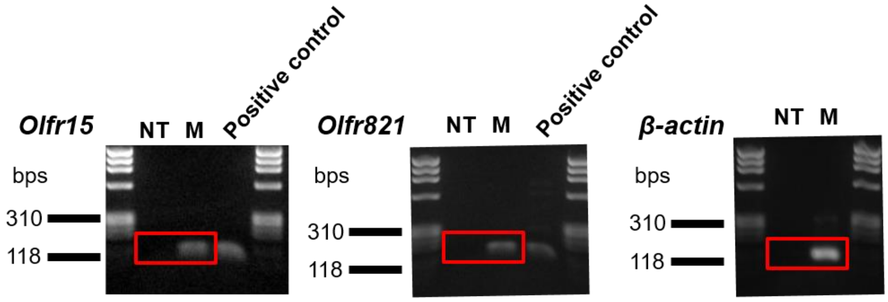

Figure 1g

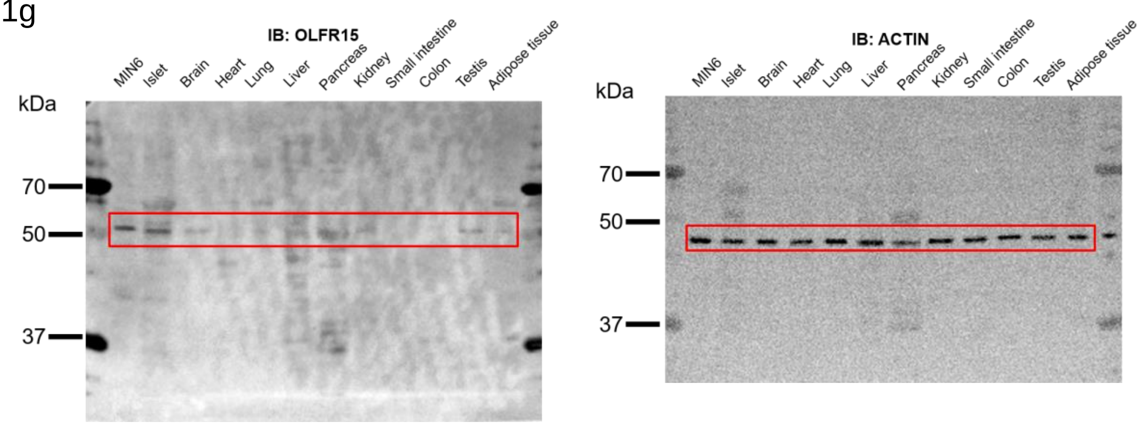

Figure 3a

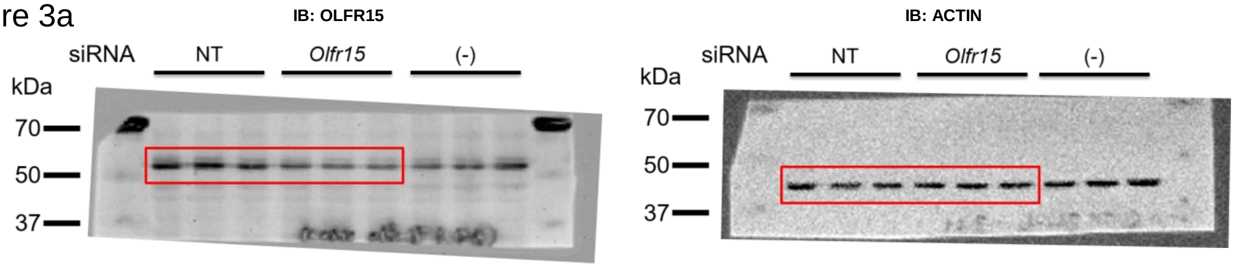

Munakata et al Supplementary Table S1

Supplementary Table S1: Expression levels of olfactory receptors detected both in murine pancreatic islets and in MIN6 cells, as determined by microarray analysis.

Olfactory receptor	Murine pancreatic islets raw signal intensity	MIN6 cells raw signal intensity
Olfr15	20.920343	130.120878
Olfr53	22.946848	80.034925
Olfr59	30.220272	20.017795
Olfr103	33.03314	121.69553
Olfr123	37.94607	106.642707
Olfr128	127.86343	220.11958
Olfr139	18.844282	36.9536225
Olfr149	34.058666	127.371565
Olfr159	20.959398	67.447168
Olfr166	40.251144	57.503086
Olfr187	36.16885	438.018165
Olfr297	15.73172	69.7502885
Olfr373	11.388631	1464.9468
Olfr456	13.785051	42.820622
Olfr469	14.20941	62.2730385
Olfr513	13.51648	78.1911875
Olfr521	91.30448	263.046625
Olfr522	18.569136	581.06604
Olfr524	16.59657	165.037765
Olfr544	62.99822	5743.4537
Olfr549	154.96353	574.72705
Olfr559	38.89793	35.5667745
Olfr609	33.25143	159.18508
Olfr611	351.15707	851.689975
Olfr656	25.083834	27.686277
Olfr661	70.22304	44.096372
Olfr738	15.87997	171.1226
Olfr745	129.5192	1783.6087
Olfr780	50.426838	45.249792
Olfr821	44.370987	456.59856
Olfr978	17.94686	26.5334155
Olfr1030	23.842377	70.434433
Olfr1043	10.716141	32.5003375
Olfr1131	30.606197	60.88681
Olfr1134	15.036688	52.7861385
Olfr1143	25.22518	261.86817
Olfr1170	13.81694	45.605062
Olfr1198	61.393494	33.170029
Olfr1222	163.90225	126.715708
Olfr1344	15.882741	17.811113
Olfr1350	41.718533	225.42141
Olfr1384	242.84653	111.953368
Olfr1393	86.42011	19.660792
Olfr1410	86.610985	96.44829
Olfr1417	43.24698	32.9325865
Olfr1441	76.85096	38.428495
Olfr1443	56.53103	156.70206

Supplementary Table S2: The oligonucleotide primers used for RT-PCR.

	forward (5' to 3')	reverse (5' to 3')
**rat** ***β-actin***	GCTGCTCACCGAGGC	CTCGGTCAGGATCTTCAT
Olr1356	CTGTGTGAGGTACCCGCCATG	TCAGCTGGCTCCTCTTCCTTTC
**human** ***β-ACTIN***	AAGGATTCCTATGTGGGC	CATCTCTTGCTCGAAGTC
OR2C1	ATGGACGGGGTGAATGATAG	TCAGCCAACTTCTCTTCC
**murine** ***β-actin***	GGTCAGAAGGACTCCTATGT	ATGAGGTAGTCTGTCAGGTC
Olfr15	ATGGAGGTGGACAGCAACAG	CATGGCGGGTACCTCACACAG
Olfr821	AACCACACAACGGTGACAGTA	CATGTATGTGAGAAAGAGAAGGAC
Olfr1222	ACTAGCCACTTCTTTGCAGGAGTT	CCAGGCCACCATTACGAGAG
$G\alpha_{olf}$	AACGAGTACCAGCTGATCGACTG	TCACAAGAGTTCGTACTGCTTGAG
Omp	GACCTCACCAACCTCATGACAC	GCGTCTGCCTCATTCCAATC

Supplementary Table S3: The oligonucleotide primers used for real-time RT-PCR.

	forward (5' to 3')	reverse (5' to 3')
***β*-actin**	GATGCCCTGAGGCTCTT	TGTGTTGGCATAGAGGTCTTTAC
Plc-β1	AAGCCAGATGGAAGAGGAGA	GCGGATCTCATTGTGTTTCT
Plc-β3	TCCTTTGAGAACCATGTCG	ATTCTTCACCAGGATACGGC
Plc-β4	CCCTGACAACGGATCACA	GTGCAGATCCCGGATTTC
Plc-γ1	GACTTCTCGGGACTTTGACC	GTTAAGCCCTTGATCCACAT
Plc-δ4	AGACCAGCCATGTGGAGAAC	CAGGTCCAAGGTAGGGTGTA
Gnaq	CAGACGACGGGAATATCAGT	AGATAGGAAGGGTCGGCTACA
